# Implementation of a performance-based financing scheme in Malawi and resulting externalities on the quality of care of non-incentivized services

**DOI:** 10.1186/s12884-021-03880-9

**Published:** 2021-05-29

**Authors:** Stephan Brenner, Caterina Favaretti, Julia Lohmann, Jobiba Chinkhumba, Adamson S. Muula, Manuela De Allegri

**Affiliations:** 1 Heidelberg Institute of Global Health, Heidelberg University Hospital and Medical Faculty, University of Heidelberg, INF 130.3, 69120 Heidelberg, Germany; 2 Department of Global Health and Development, London School of Hygiene and Tropical Medicine, London, UK; 3 Department of Health Systems and Policy, Health Economics and Policy Unit, University of Malawi College of Medicine, Blantyre, Malawi; 4 School of Public Health and Family Medicine and ACEPHEM, University of Malawi College of Medicine, Blantyre, Malawi

**Keywords:** Performance-based financing, Antenatal care, Quality of care, Externalities, Stepped implementation

## Abstract

**Background:**

Countries in Africa progressively implement performance-based financing schemes to improve the quality of care provided by maternal, newborn and child health services. Beyond its direct effects on service provision, evidence suggests that performance-based financing can also generate positive externalities on service utilization, such as increased use of those services that reached higher quality standards after effective scheme implementation. Little, however, is known about externalities generated within non-incentivized health services, such as positive or negative effects on the quality of services within the continuum of maternal care.

**Methods:**

We explored whether a performance-based financing scheme in Malawi designed to improve the quality of childbirth service provision resulted positive or negative externalities on the quality of non-targeted antenatal care provision. This non-randomized controlled pre-post-test study followed the phased enrolment of facilities into a performance-based financing scheme across four districts over a two-year period. Effects of the scheme were assessed by various composite scores measuring facilities’ readiness to provide quality antenatal care, as well as the quality of screening, prevention, and education processes offered during observed antenatal care consultations.

**Results:**

Our study did not identify any statistically significant effects on the quality of ANC provision attributable to the implemented performance-based financing scheme. Our findings therefore suggest not only the absence of positive externalities, but also the absence of any negative externalities generated within antenatal care service provision as a result of the scheme implementation in Malawi.

**Conclusions:**

Prior research has shown that the Malawian performance-based financing scheme was sufficiently effective to improve the quality of incentivized childbirth service provision. Our findings further indicate that scheme implementation did not affect the quality of non-incentivized but clinically related antenatal care services. While no positive externalities could be identified, we also did not observe any negative externalities attributable to the scheme’s implementation. While performance-based incentives might be successful in improving targeted health care processes, they have limited potential in producing externalities – neither positive nor negative – on the provision quality of related non-incentivized services.

**Supplementary Information:**

The online version contains supplementary material available at 10.1186/s12884-021-03880-9.

## Background

High quality antenatal care (ANC) visits throughout pregnancy reduce both stillbirth rate [1] and adverse maternal and newborn outcomes [2]. ANC ideally consists of an initial client assessment in early pregnancy followed by several follow-up visits to ensure continuous clinical monitoring and integrated care for mother and baby [3]. Since the mid-2000s, ANC has been implemented as an essential component of the “continuum of care for mothers and newborns in Sub-Saharan Africa” (SSA) [3]. This continuum of care aims at providing uninterrupted care to women and their children throughout pregnancy, childbirth and beyond, with ANC serving as initial contact point between a pregnant woman’s and the health system. Operationalization of ANC as initial entry point to continued care remains challenging. By 2017, for instance, coverage of pregnant women with at least four repeated ANC visits in SSA ranged only between 25 and 76% with most ANC providers meeting only relatively low quality of care standards [4].

To improve these shortcomings in the provision of ANC and continued maternal and newborn healthcare (MNH), many countries in SSA have started implementing different types of performance-based incentives in recent years [5]. Performance-based financing (PBF), as a particular form of performance-based incentives, refers to provider payments linked to the achievement of preset service quantity and quality targets [6]. PBF payments for ANC visits have shown positive effects on the quality of essential healthcare during pregnancy, such as blood pressure screening, tetanus immunization, or malaria prevention [7–9].

Little, however, is known about the extent to which effects in the quality of a PBF-incentivized service affect the quality of other services offered by the same provider or facility. PBF schemes are usually designed to simultaneously incentivize performance across a number of provided services, especially when used to improve service provision within the maternal care continuum. Thus, the assessment of external effects, or externalities, on the provision of non-incentivized services is usually almost impossible. Evidence from PBF evaluations indicate the existence of positive externalities on health-seeking behavior with patients’ preferences towards use of PBF-incentivized services [7, 8]. Furthermore, some evidence also suggests the existence of negative externalities, especially in form of unintended effects, such as providers’ neglect of non-incentivized services [10]. So far, existing evidence on the extent to which PBF incentives might generate external effects on non-incentivized services, especially with regard to services within the continuum of care, remains inconclusive.

Malawi represents one of many SSA countries which has implemented PBF schemes to improve MNH care provision. One particular scheme, the *Results-Based Financing For Maternal and Newborn Health* (RBF4MNH) Initiative, was introduced in 2013. It was designed to specifically improve the quality of childbirth care provided at enrolled health facilities in response to the country’s high maternal mortality ratio (estimated at 675 deaths/100,000 livebirths at that time) [11]. The RBF4MNH design did not directly incentivize ANC or any other maternal care services within the continuum. This particular scheme therefore provides an opportunity to explore whether performance-based incentives to a particular group of healthcare providers, namely midwives attached to a defined set of clinical services, namely facility-based childbirth, might have external benefits to other non-incentivized maternal care services.

The RBF4MNH scheme in Malawi therefore offers a unique opportunity to explore the existence of externalities generated by a PBF implementation on the provision of non-incentivized services within the maternal care continuum. To further expand existing evidence, our study assesses the extent performance-based incentives linked to maternal healthcare workers’ performance in childbirth care resulted in observable externalities on these health workers’ performance in ANC provision.

## Methods

### Study context

In Malawi, maternal health services, including ANC, are mostly provided through public (government-owned) as well as private not-for-profit (faith-based organizations) sectors [12]. By 2013, about 90% of hospitals and health centers offered ANC services [13]. In 2010, ANC coverage was rather low with only 46% of pregnant women attending at least four ANC visits and only 12% starting ANC during the first pregnancy trimester [11]. Nevertheless, ANC content was found to be of relatively high quality with 84% of women reporting blood pressure screening, 91% iron deficiency prevention, and 80% education on danger signs [11].

In April 2013, the Ministry of Health launched a PBF scheme, the RBF4MNH Initiative, in four districts (Balaka, Dedza, Mchinji, Ntcheu) to improve quality and utilization of facility-based childbirth [14, 15]. District selection was non-random and reflected the Ministry’s decision to first enroll districts with comparatively weaker maternal health indicators with respect to maternal and newborn service coverage and mortality outcomes. All districts are located in Malawi’s Central Region, except for Balaka, which is located in the Southern Region. In 2010, ANC use in these four was comparably high ranging from 90% in Mchinji to 95% in Dedza [11].

### RBF4MNH implementation and related evidence

The RBF4MNH scheme consisted of two components: (1) performance-based payments to facilities and district health management teams linked to defined delivery care quality targets (of note, no ANC-specific targets or incentives); and (2) an additional demand-side component with conditional cash transfers to pregnant women to the use of childbirth services (including a 48-h postpartum stay) at their respective PBF catchment facility (of note, enrolment of women into the demand component occurred during preceding ANC visits) [14, 15].

RBF4MNH implantation occurred in two phases. Initially (phase 1 starting April 2013), 18 non-randomly selected EmOC facilities (four hospitals, 14 health centers) across the four districts enrolled in the scheme. In October 2014 (start of phase 2), 15 additional EmOC facilities (three hospitals, 12 health centers) within the same four districts enrolled. All enrolled facilities received performance-based payments in addition to their usual budget allocations. Quarterly payment amounts reflected facilities’ achievement towards defined childbirth-related performance targets measured by a total of six quantity indicators. These amounts could be further deflated for facilities that under-performed with regard to childbirth quality aspects measured by seven quality indicators. Similar sets of childbirth related quantity and quality indicators were used to reimburse the performance of district health managers. Portions of quarterly payments earned by both facilities and district health managers could be allocated as salary bonuses to individual staff members based on their respective performance contributions. As part of the PBF component, facilities also received some upfront inputs in form of minor infrastructure repairs or essential equipment procurement, such as renovation of labor rooms, purchase of disinfectants, and replacement of blood pressure machines.

Existing evidence related to RBF4MNH implementation suggests significant positive effects of the scheme on the clinical quality of childbirth care, mostly by increasing health workers’ adherence to technical standards as well as through improved supply chain management [16]. Of note, the RBF4MNH did not produce external effects on service coverage, neither with respect to for women’s use of facility-based childbirth care, nor ANC [17, 18].

### Study rationale and design

Understanding externalities with respect to PBF implementation is of relevance, as PBF schemes are increasingly viewed as potential drivers in reforming a country’s health service provision systems [19]. To this end, the economic concept of externalities describes any additional gains or drawbacks produced by an activity, program or policy. For the purpose of our study, we apply the term externality to refer to any observable positive or negative effects generated by the RBF4MNH scheme with respect to the quality of ANC service provision.

Given the above improvements on some aspects of childbirth care, our expectation was to observe positive externalities of the scheme to ANC provision, such as adherence to ANC standards and availability of ANC-specific supplies. This expectation was further supported by the fact that (1) the maternal health workers incentivized by the RBF4MNH for providing quality childbirth (i.e. midwives) also provided ANC services, (2) upfront investments provided to enrolled facilities through the RBF4MNH improved maternal care inputs and infrastructure relevant to childbirth as well as ANC provision, and (3) observed improvements in the procurement and supply of consumables and medicines for intrapartum care likely had some beneficial effects on consumables used in ANC provision.

To assess any externalities to ANC service quality, this study followed a non-randomized controlled pre-post-test design including a total of 33 maternal care facilities across the four study districts. This design was nested into a larger impact evaluation assessing the RBF4MNH effect on MNH service utilization and quality [20]. The design accounted for the stepwise implementation with initially 18 PBF-enrolled facilities (four hospitals, 14 health centers) serving as ‘phase 1’ group, followed by five facilities (two hospitals, three health centers) as ‘phase 2’ group after scheme expansion in 2014. The remaining ten facilities (one hospital, nine health centers) served as ‘no PBF’ group. Data were collected at three time points: baseline (April–May 2013, before official program launch), midline (June–July 2014, approximately one year after program launch) and endline (June–July 2015, approximately two years after program launch).

### Sampling and data collection

Using direct observation, data were collected on both facilities’ readiness to provide key inputs to ANC service provision and ANC case management. To do so, we relied on two nested study samples: the facility sample described above and a sub-sample of ANC cases observed at each of the sampled facilities. Facility observations were conducted by trained research assistants using a structured facility inventory checklist. At each sampled facility the inventory checklist was used to collect information on structural and infrastructural aspects related to ANC service provision, including the availability and accessibility of essential drugs, supplies, and functional equipment. ANC case observations were conducted by trained research assistants recording provider-patient interactions during individual ANC consultations using a structured case management checklist. This checklist recorded information on provider’s adherence to clinical ANC standards as defined by the Malawi Performance Quality Improvement program [21], including patient history assessment, physical examination, laboratory screening, preventive measures and educational content. ANC case sampling occurred during antenatal clinic days and followed a convenience approach: after completing the observation of the first identified case of the day, research assistants consistently enrolled the next-in-line patient who consented participation in the clinical observation. This approach was repeated after each completed observation until a total sample of five observation per facility was reached. This approach did not entail proportional or stratified sampling steps and did not account for specific case characteristics.

### Outcome variables

The World Health Organization (WHO) defines ANC as healthcare intervention for pregnant women intended to prevent adverse health outcomes for mother and baby during and following pregnancy [22]. In essence, ANC comprises three key components: (1) identification of risk factors associated with negative pregnancy outcomes, including. Screening of clinical signs and symptoms related to pregnancy- and birth-related complications; (2) control of pregnancy-related disorders that negatively affect the mother or baby, including. Prevention of common pregnancy risks related to infectious, metabolic, or obstetric causes; and (3) information, education and counselling of the parents on relevant pregnancy, birth, and postnatal health issues, including promotion of health and health-seeking behaviors [22]. In defining quality of care outcome measures, we focused on inputs and processes related to those three components.

Quality measures were conceptually rooted in Donabedian’s framework for the assessment of quality of care [23]. Using the *WHO Recommendations on Antenatal Care for a Positive Pregnancy Experience* [22] and the *Service Availability and Readiness Assessment Reference* Manual [24] as our basis for indicator selection, we identified a set of 15 input variables measuring facility readiness from the facility inventory dataset and 56 process variables related to quality ANC provision from observation dataset (see “ANC Score and Indicator Matrix” in Additional file 1 for more details). All variables consisted of binary data and were further combined into six composite variables as outlined in Table 1: *ANC Readiness*, which we computed at the facility level, *Screening First Visit*, *Screening Follow-up*, *Prevention First Visit*, *Prevention Follow-up*, *Information & Education*, all of which we computed at the case level.

**Table 1:** Overview and definitions of outcome variables

Outcome variable	Analytical level	ANC quality aspects reflected
**ANC Readiness** (15 items)	Facility	Structural readiness, staff & equipment, medications & supplies, diagnostic tests
**Screening First Visit Cases** (31 items)	Case	Focused history (including obstetric, pregnancy, medical, and social history), focused exam (including vital sign assessment, physical check-up, diagnostic testing)
**Screening Follow-up Cases** (17 items)	Case	Focused history (including pregnancy and social history, review of ANC patient record for obstetric and social history), focused exam (including vital sign assessment, physical check-up)
**Prevention First Visit Cases** (11 items)	Case	Prescription or administration of drugs and supplies (including iron/folate supplements, correct IPTp administration in respect to trimester, tetanus vaccination, provision of insecticide-treated bed net)
**Prevention Follow-up Cases** (9 items)	Case	Prescription or administration of drugs (including iron/folate supplements, correct IPTp administration in respect to trimester, tetanus vaccination),
**Information & Education** (10 items)	Case	Counselling of patient (including explanation of clinical findings, discussion of health-seeking for danger signs, diet, birth & preparedness planning, postpartum contraception, breastfeeding, encourages question)

ANC = antenatal care, IPTp = intermittent preventive treatment of malaria in pregnancy

Screening and prevention composites were computed separately for first ANC visit and follow-up cases, since respective clinical content differs slightly as per above national and international standards, which propose a more detailed assessment and work-up for first visit cases. Composites were computed by additive aggregation of all equally weighted items identified for each outcome. To facilitate comparison, composite values were further transformed to range between 0 and 1 based on the underlying value range of each outcome variable [25].

### Analytical approach

We relied on simple descriptive statistics to compare the distribution of facilities and cases by implementation group (i.e. phase 1, phase 2, and no PBF) for each time point. We used Fisher’s exact test and analysis of variance to assess differences in key characteristics between intervention groups at baseline. To estimate the RBF4MNH program’s effect on each ANC outcome variable, we employed a mixed effects multi-level linear regression model. The longitudinal nature of our data with three data points for each facility over time enabled us to further control for fixed effects at the facility level. In modelling effects on outcome variables computed at the case level, we further clustered standard errors at the facility level (i.e. the level of treatment assignment). The resulting model specification for case-level outcomes is illustrated by the following equation:

$$ {Y}_{ift}={\alpha}_f+\beta {T}_t+\gamma {Phase}_{ift}+\delta {X}_{it}+{u}_{it}+{\varepsilon}_{ift} $$

where *Y* represents each measured outcome for case *i* at facility cluster *f* at time *t*, *T* a categorical variable indicating the time point of data collection (0 = baseline, 1 = midline, 2 = endline), and *Phase* a categorical variable indicating the implementation phase in which a given facility enrolled under the PBF scheme (0 = never, 1 = ‘phase 1’-enrolment, 2 = ‘phase 2’-enrolment). Coefficient *γ* therefore represents the PBF-attributable effect, *α* the facility fixed effect, *β* the effect of time, *δ* the effects of additional co-variates *X* (see below) used in each model, *u* the random effect of individual cases, and *ε* the error term. For the outcome ‘ANC Readiness’ based on measurements obtained at the facility level only, the above model was reduced to only include the fixed effects *α* and *β*, but not the random case-level effect *u*.

As additional co-variates to the above case-level models, we identified variables expected to affect observed quality scores independently of the PBF schemes. These included, case consulted by an ANC-qualified vs. non-qualified provider, defining whether a provider belongs to any professional cadre considered skilled birth attendant in Malawi [26] and assuming a positive effect on process outcomes if provider is more qualified, and consultation duration lasting more vs. less than 13 min, with this cut-off defined by the sample mean, assuming longer consultation times allow increase chance of better-quality outcomes.

## Results

### Sample distribution

Table 2 summarizes the distribution of the studied health facilities by district, facility type, and ownership for each implementation group at each time point. At baseline, we were able to collect complete information for only 30 out of the 33 sampled facilities. For one health center in the ‘no PBF’ and two in the ‘phase 1’ group available readiness data were incomplete. At midline for only 32, as data were incomplete for one ‘no PBF’ health center. Following the RBF4MNH implementation process, about half of facilities belonged to the ‘phase 1’ group. Within implementation groups, distribution of studied facilities across districts was approximately even. The majority of facilities in each implementation group included health centers and public facilities, however, this was not the case for the ‘phase 2’ group with a slightly higher proportion of private facilities.

**Table 2:** Distribution of key characteristics across sampled health facilities by implementation group and time point

	Baseline			Midline (Year 1)			Endline (Year 2)		
	No PBF	Phase 1	Phase 2	No PBF	Phase 1	Phase 2	No PBF	Phase 1	Phase 2
**Number (% total) studied facilities:**	9 (30.0)	16 (53.3)	5 (16.7)	9 (28.1)	18 (56.3)	5 (15.6)	10 (30.3)	18 (54.6)	5 (15.1)
***By district, n (%)***									
Balaka	2 (22.2)	4 (25.0)	2 (40.0)	2 (22.2)	4 (22.2)	2 (40.0)	2 (20.0)	4 (22.2)	2 (40.0)
Dedza	3 (33.4)	3 (18.8)	1 (20.0)	3 (33.4)	4 (22.2)	1 (20.0)	3 (30.0)	4 (22.2)	1 (20.0)
Mchinji	2 (22.2)	4 (25.0)	1 (20.0)	2 (22.2)	5 (27.8)	1 (20.0)	2 (20.0)	5 (27.8)	1 (20.0)
Ntcheu	2 (22.2)	5 (31.2)	1 (20.0)	2 (22.2)	5 (27.8)	1 (20.0)	3 (30.0)	5 (27.8)	1 (20.0)
***By facility type, n (%)***									
Health center	8 (88.9)	12 (75.0)	3 (60.0)	8 (88.9)	14 (77.8)	3 (60.0)	9 (90.0)	14 (77.8))	3 (60.0)
Hospital	1 (11.1)	4 (25.0)	2 (40.0)	1 (11.1)	4 (22.2)	2 (40.0)	1 (10.0)	4 (22.2)	2 (40.0)
By facility ownership, n (%)*									
Public	6 (66.7)	15 (93.8)	2 (40.0)	6 (66.7)	17 (94.4)	2 (40.0)	7 (70.0)	17 (94.4)	2 (40.0)
Private non-profit	3 (33.3)	1 (6.2)	3 (60.0)	3 (33.3)	1 (5.6)	3 (60.0)	3 (30.0)	1 (5.6)	3 (60.0)

* Distribution at baseline statistically significantly different between facility groups (p < 0.05) based on Fisher’s exact test

Table 3 summarizes the distribution of studied ANC cases by key facility-specific (i.e. district, facility type, ownership) and case-specific (i.e. visit type, provider qualification, consultation time) characteristics for each implementation group and time point. Facility distribution across groups influenced case distribution. We observed a total of 243 cases at baseline, 301 at midline, and 289 at endline, with the majority of cases observed in the ‘phase 1’ group. Distribution of cases between districts was approximately even within each group. Almost all cases were attended by a qualified ANC provider regardless of implementation group. At baseline, average consultation time was statistically significant shorter in the ‘phase 2’ group.

**Table 3:** Distribution of key characteristics across sampled antenatal care cases by implementation group and time point

	Baseline			Midline (Year 1)			Endline (Year 2)		
	No PBF	Phase 1	Phase 2	No PBF	Phase 1	Phase 2	No PBF	Phase 1	Phase 2
**Number (% total) observed cases:**	47 (19.3)	194 (61.2)	47 (19.3)	65 (21.6)	174 (57.8)	62 (20.6)	65 (22.5)	178 (61.6)	46 (15.9)
***By district, n (%)***									
Balaka	8 (17.0)	32 (21.5)	18 (38.3)	12 (18.5)	22 (12.6)	25 (40.3)	14 (21.5)	38 (21.4)	18 (39.1)
Dedza	16 (34.0)	35 (23.5)	12 (25.5)	25 (38.4)	50 (28.7)	19 (30.7)	15 (23.1)	44 (24.7)	12 (26.1)
Mchinji	13 (27.7)	39 (26.2)	10 (21.3)	15 (23.1)	74 (42.5)	10 (16.1)	8 (12.3)	37 (20.8)	5 (10.9)
Ntcheu	10 (21.3)	43 (28.8)	7 (14.9)	13 (20.0)	28 (16.1)	8 (12.9)	28 (43.1)	59 (33.1)	11 (23.9)
By facility type, n (%)*									
Health center	44 (93.6)	99 (66.4)	20 (42.5)	60 (92.3)	79 (45.4)	34 (54.8)	59 (90.8)	97 (54.5)	25 (54.4)
Hospital	3 (6.4)	50 (33.6)	27 (57.5)	5 (7.7)	95 (54.6)	28 (45.2)	6 (9.2)	81 (45.5)	21 (45.6)
By facility ownership n (%)*									
Public	33 (70.2)	139 (93.3)	10 (21.3)	47 (72.3)	171 (98.3)	24 (38.7)	40 (61.5)	171 (96.1)	20 (43.5)
Private non-profit	14 (29.8)	10 (6.7)	37 (78.7)	18 (27.7)	3 (1.7)	38 (61.3)	25 (38.5)	7 (3.9)	26 (56.5)
By ANC visit type, n (%)*									
First Visit	31 (66.0)	71 (47.6)	39 (83.0)	28 (43.1)	57 (32.8)	24 (38.7)	19 (29.2)	64 (36.0)	31 (67.4)
Follow-up Visit	16 (34.0)	78 (52.4)	8 (17.0)	37 (56.9)	117 (67.2)	38 (61.3)	46 (70.8)	114 (64.0)	15 (32.6)
***By ANC provider qualification*** ^***a***^***, n (%)***									
Qualified	47 (100)	147 (99.7)	47 (100)	59 (90.8)	115 (66.1)	49 (79.0)	65 (100)	138 (77.5)	46 (100)
Not qualified	0 (0)	2 (1.3)	0 (0)	6 (9.2)	59 (33.9)	13 (21.0)	0 (0)	40 (22.5)	0 (0)
Duration (in minutes) of consultation, mean (SD)*	6.8 (3.6)	7.1 (4.0)	4.6 (3.5)	6.8 (3.6)	7.1 (4.0)	4.6 (3.5)	8.8 (6.1)	12.0 (8.8)	8.4 (7.2)

^*a*^ *“Qualified” category includes the following: registered nurse midwife, enrolled nurse midwife, enrolled nurse, enrolled midwife, medical assistant, clinical health officer, general physician, obstetrician*

* Distribution at baseline statistically significantly different between implementation groups (p < 0.05) based on Fisher’s exact test for categorical, and ANOVA for continuous variables

### ANC scores and effect estimates

Table 4 outlines the average score distributions of the four outcome variables for each implementation group at each time point. Average service readiness scores ranging between 0.61 to 0.80 points were overall higher than process quality scores. The least available items included tests for hemoglobin, urine, and syphilis independent of implementation group (data not shown).

**Table 4:** Distribution of average scores for each outcome variable by implementation group and time point and resulting estimated effect sizes

	Baseline			Midline (Year 1)			Endline (Year 2)			PBF-attributable Effect	
	No PBF	Phase 1	Phase 2	No PBF	Phase 1	Phase 2	No PBF	Phase 1	Phase 2		
	***mean (SD)***	***mean (SD)***	***mean (SD)***	***mean (SD)***	***mean (SD)***	***mean (SD)***	***mean (SD)***	***mean (SD)***	***mean (SD)***	***Effect size (95%-CI)***	
**ANC Service Readiness** ^**a**^ *	0.61 (0.15)	0.63 (0.13)	0.80 (0.10)	0.65 (0.24)	0.69 (0.15)	0.67 (0.11)	0.65 (0.14)	0.79 (0.17)	0.71 (0.15)	0.06	(−0.13; 0.07)
**ANC Screening Quality** ^**b**^											
First Visit *	0.29 (0.15)	0.27 (0.12)	0.26 (0.17)	0.34 (0.16)	0.46 (0.15)	0.29 (0.13)	0.41 (0.16)	0.43 (0.16)	0.31 (0.16)	0.07	(−0.01; 0.16)
Follow-up visit	0.19 (0.18)	0.32 (0.23)	0.64 (0.23)	0.49 (0.29)	0.55 (0.25)	0.23 (0.20)	0.38 (0.28)	0.44 (0.24)	0.27 (0.26)	0.13	(−0.35; 0.19)
**ANC Prevention Quality** ^**b**^											
First Visit	0.83 (0.20)	0.68 (0.24)	0.69 (0.27)	0.47 (0.27)	0.61 (0.28)	0.57 (0.29)	0.72 (0.33)	0.65 (0.27)	0.62 (0.27)	0.06	(−0.09; 0.21)
Follow-up visit	0.52 (0.20)	0.52 (0.23)	0.68 (0.13)	0.51 (0.31)	0.49 (0.22)	0.38 (0.24)	0.48 (0.26)	0.50 (0.23)	0.30 (0.30)	0.05	(−0.09; 0.19)
**ANC Information & Education Quality** ^**b**^	0.48 (0.28)	0.35 (0.25)	0.34 (0.28)	0.47 (0.23)	0.45 (0.23)	0.41 (0.19)	0.47 (0.29)	0.54 (0.29)	0.32 (0.28)	−0.02	(−0.18; 0.13)

*CI = confidence interval*

^*a*^ *score computed at facility level;* ^*b*^ *score computed at case level*

* Distribution at baseline statistically significantly different between implementation groups (p < 0.05) based on ANOVA

Compared to prevention and information/education quality, average screening scores were overall lowest, but followed an upward trend over time, except for follow-up visits in the ‘phase 2’ group which trended downward. Especially at midline, we observed about a 0.2-point (or 20%-point) increase from baseline in the ‘phase 1’ group. This upward trend was largely the result of more frequently observed assessments of pre-eclampsia symptoms in the patient history, while other processes closely related to the RBF4MNH incentives remained largely unchanged, such as HIV screening, physical assessment of pre-eclampsia signs including blood pressure, and routine hand-hygiene prior to physical contact with the patient (data not shown).

In comparison to screening and information/education quality, average prevention scores were overall higher. Especially in the ‘phase 2’ group, we observed a pronounced drop in this score at midline for the follow-up category. This drop was largely driven by a strong decline in the frequency of observed iron-sulphate provision, which was paralleled by an isolated drop in iron sulphate availability across facilities, but more pronounced in the ‘phase 2’ group (data not shown).

Average information quality remained rather unchanged over time, except for the ‘phase 1’ group, which showed an upward trend in scores. This trend was largely driven by a more frequently observed sharing of information related to danger signs and birth preparedness planning (data not shown). At baseline, average scores did not differ statistically between implementation groups, except for ‘ANC Readiness’, which demonstrated a significantly higher score for ‘phase 2’ facilities, and Screening Quality of first-visit cases.

In the last column of Table 4, we report the effect sizes with confidence intervals for each ANC outcome variable estimated by our models. Overall, we did not observe any statistically significant effects attributable to the RBF4MNH scheme with regard to both service readiness and quality of care outcomes.

## Discussion

This study analyzed potential externalities of a PBF scheme, measured in relation to the quality of non-incentivized services along the MNH care continuum. The RBF4MNH scheme design took a relatively narrow implementation focus on the quality and utilization of facility-based deliveries only. This offered a unique opportunity to explore potential externalities generated by the scheme’s performance-based financial incentives on input and process aspects of non-targeted ANC service quality.

In this study, we did not find evidence that the RBF4MNH scheme produced either positive or negative externalities on ANC service provision. This is relevant in so far, as our findings neither suggest an improvement nor a neglect of non-incentivized services provided by the same health worker cadre. Our findings contradict some of the existing literature suggesting that PBF can result in positive quality improvements across different services along the MNH continuum [7, 27]. Nonetheless, we wish to draw the reader’s attention to the fact that we found no evidence of negative externalities on non-incentivized services provided by the same PBF-incentivized health staff. This is in contrast to concerns raised by some authors with respect to PBF implementation [10],

While our expectation was to identify some improvements in the quality of ANC attributable to the RBF4MNH, this was not the case. One explanation for the lack of positive externalities might be that the RBF4MNH scheme was designed with a focus on childbirth, especially on the provision of emergency obstetric care [15]. There was no explicit emphasis on designing performance incentives to purposefully link utilization of high-quality ANC to emergency obstetric care outputs. More research is therefore needed with respect to PBF scheme implementation and evaluation as a means to improve service provision within the maternal care continuum. More detailed insight is needed to understand how performance incentives should be implemented to better link or complement quality gains across services.

Another explanation for the absence of observed externalities in our study might be the nested implementation of the RBF4MNH scheme in the four districts. With respect to childbirth service use, this phased enrolment did produce shifts in demand with pregnant women residing in catchment areas of non-enrolled facilities seeking care at PBF facilities [28]. Of note, this observed shift in demand was not only driven by users directly, but also by maternal care providers from non-enrolled faculties, who referred pregnant patients with risk factors to receive their care from RBF facilities [28]. Increased utilization of PBF supported delivery services further increased the workload of maternal care workers at these facilities beyond capacity [29]. This unintended erosion of workload capacity might not only have limited the RBF4MNH’s actual potential on improving incentivized processes of care, but also its potential to produce substantial externalities in related care services.

### Methodological limitations

Our study is challenged by a number of limitations. Our findings are based on a non-experimental design with a non-random distribution of some of the observed key facility and case characteristics (Tables 2 and 3). To control for this selection bias, we adjusted our models for those observed key characteristics identified as potential confounders. The number of facility clusters (*n* = 33) was relatively small, thus reducing the statistical power to attribute small effect sizes to the RBF4MNH (i.e. type II error). With regard to our case observations, the Hawthorne effect might have biased observed performance [30]. However, this effect is understood to bias performance to be higher than usual and constant over time. With our observed ANC care provision being overall rather low, this effect would have overestimated the typical standard of care, and did probably not substantially affect the validity of our findings in this study. The overall study period of about two years might have been too short to capture the full potential of a more matured RBF4MNH implementation. Especially for facilities enrolled during phase 2, additional data points might have provided a more accurate estimate of the intervention impact on ANC quality. Lastly, the generalizability of our findings is probably fairly restricted to both our study context and the specifics of the studied RBF4MNH scheme. Any generalizations of these findings to differently designed incentive schemes operating in a different health system, population or geographical context might therefore be limited.

## Conclusions

In conclusion, our findings on observed externalities on ANC service provision in response to the RBF4MNH initiative in Malawi demonstrate that financial incentives had very limited potential to produce positive externalities on clinically relevant, but non-incentivized health service provision. An important finding of this study, however, is the absence of negative externalities with respect to ANC provision. Further research will be needed not only to more explicitly examine the actual potential of performance-based incentives to produce externalities, but also to provide better understanding how PBF schemes can be designed and implemented to ensure the quality and quantity of services provided along the MNH continuum can be successfully aligned and linked.

## Supplementary Information

**Additional file 1.** ANC Score and Indicator Matrix

## Data Availability

The datasets used and/or analyzed during the current study are available from the corresponding author on reasonable request.

## Abbreviations

ANC: Antenatal Care
MNH: Maternal and Newborn Healthcare
PBF: Performance-Based Financing
RBF4MNH: Results-Based Financing For Maternal and Newborn Health
SSA: Sub-Saharan Africa
WHO: World Health Organization

## References

[1] Menezes EV, Yakoob M, Soomro T, Haws RA, Darmstadt GL, Bhutta ZA (2009). Reducing stillbirths: prevention and management of medical disorders and infections during pregnancy. BMC Pregnancy Childbirth.

[2] Bale JR, Stoll BJ, Lucas AO, Institute of Medicine (2003). Improving Birth Outcomes: Meeting the Challenge in the Developing World.

[3] The Partnership for Maternal, Newborn and Child Health. Opportunities for Africa’s newborns: Practical data, policy and programmatic support for newborn care in Africa. Joy Lawn, Kate Kerber, editors. Cape Town, South Africa: WHO on behalf of The Partnership for Maternal Newborn and Child Health; 2006. 250 p.

[4] Kanyangarara M, Munos MK, Walker N (2017). Quality of antenatal care service provision in health facilities across sub–Saharan Africa: evidence from nationally representative health facility assessments. J Glob Health.

[5] Morgan L, Eichler R. Performance-Based Incentives in Africa. Experiences, Challenges, Lessons. Bethesda: United States Agency for International Development; 2011. (Health Systems 20/20 Project, Abt Associates Inc.).

[6] Musgrove P. Financial and other rewards for good performance or results. A Guided Tour of Concepts and Terms and a Short Glossary. In: World Bank Health Results Innovation Trust Fund. 2011.

[7] Bonfrer I, Van de Poel E, Van Doorslaer E (2014). The effects of performance incentives on the utilization and quality of maternal and child care in Burundi. Soc Sci Med.

[8] Binyaruka P, Patouillard E, Powell-Jackson T, Greco G, Maestad O, Borghi J. Effect of Paying for Performance on Utilisation, Quality, and User Costs of Health Services in Tanzania: A Controlled Before and After Study. Ostermann J, editor. PLoS One. 2015;10(8):e0135013. 10.1371/journal.pone.0135013 PMC4552688 26317510

[9] Falisse J-B, Ndayishimiye J, Kamenyero V, Bossuyt M (2015). Performance-based financing in the context of selective free health-care: an evaluation of its effects on the use of primary health-care services in Burundi using routine data. Health Policy Plan.

[10] Paul E, Albert L, Bisala BN, Bodson O, Bonnet E, Bossyns P (2018). Performance-based financing in low-income and middle-income countries: isn’t it time for a rethink?. BMJ Glob Health.

[11] Malawi National Statistical Office (2011). Malawi. Demographic and health survey 2010.

[12] Ministry of Health Malawi. Malawi Health Sector Strategic Plan 2011–2016. 2011 2016.

[13] Ministry of Health Malawi (2014). Malawi Service Provision Assessment (MSPA) 2013–14.

[14] Ministry of Health Malawi (2012). Inception Report Results Based Financing for Maternal and Neonatal Health (RBF4MNH Initiative) 2012–2014.

[15] White-Kaba M. How rewards improve health practice in Malawi: Learnings from a Maternal and Newborn Health Initiative. Bonn, Germany: Federal Ministry for Economic Cooperation and Development (BMZ); 2017. (Deutsche Gesellschaft für Internationale Zusammenarbeit (GIZ) GmbH, editor. German Health Practice Collection).

[16] Brenner S, Wilhelm DJ, Lohmann J, Kambala C, Chinkhumba J, Muula AS, et al. Implementation research to improve quality of maternal and newborn health care, Malawi. Bull World Health Organ. 2017;95(7):491-502. 10.2471/BLT.16.178202 PMC5487969 28670014

[17] Brenner S, Mazalale J, Wilhelm D, Nesbitt RC, Lohela TJ, Chinkhumba J, Lohmann J, Muula AS, de Allegri M (2018). Impact of results-based financing on effective obstetric care coverage: evidence from a quasi-experimental study in Malawi. BMC Health Serv Res.

[18] Brenner S, De Allegri M, Kambala C, Lohmann J, Moszyk D, Mazalale J, et al. Final results of the RBF4MNH impact evaluation [internet]. 2016 Available from: http://sphfm.medcol.mw/wp-content/uploads/2016/07/Final-Results-Report-1.pdf

[19] Meessen B, Soucat A, Sekabaraga C (2011). Performance-based financing. Just a donor fad or a catalyst towards comprehensive health-care reform?. Bull World Health Organ.

[20] Brenner S, Muula AS, Robyn PJ, Bärnighausen T, Sarker M, Mathanga DP, Bossert T, de Allegri M (2014). Design of an impact evaluation using a mixed methods model--an explanatory assessment of the effects of results-based financing mechanisms on maternal healthcare services in Malawi. BMC Health Serv Res.

[21] Jhpiego (2012). Maternal and child health integrated program (MCHIP) Malawi.

[22] WHO (2016). WHO recommendations on antenatal care for a positive pregnancy experience.

[23] Donabedian A (1988). The quality of care. How can it be assessed?. JAMA..

[24] WHO (2015). Service Availability and Readiness Assessment (SARA): An annual monitoring system for service delivery.

[25] OECD (2008). Editor. Handbook on constructing composite indicators methodology and user guide.

[26] Adegoke A, Utz B, Msuya SE, van den Broek N (2012). Skilled birth attendants: who is who? A descriptive study of definitions and roles from nine sub Saharan African countries. PLoS One.

[27] Basinga P, Gertler PJ, Binagwaho A, Soucat AL, Sturdy J, Vermeersch CM (2011). Effect on maternal and child health services in Rwanda of payment to primary health-care providers for performance: an impact evaluation. Lancet.

[28] De Allegri M, Brenner S, Kambala C, Mazalale J, Muula AS, Chinkhumba J (2020). Exploiting the emergent nature of mixed methods designs: insights from a mixed methods impact evaluation in Malawi. Health Policy Plan.

[29] Lohmann J, Wilhelm D, Kambala C, Brenner S, Muula AS, De Allegri M (2018). ‘The money can be a motivator, to me a little, but mostly PBF just helps me to do better in my job.’ An exploration of the motivational mechanisms of performance-based financing for health workers in Malawi. Health Policy Plan.

[30] McCarney R, Warner J, Iliffe S, van Haselen R, Griffin M, Fisher P (2007). The Hawthorne effect: a randomised, controlled trial. BMC Med Res Methodol.

