# Supplementary material for: Implementation of a performance-based financing scheme in Malawi and resulting externalities on the quality of care of non-incentivized services
Source: BMC Pregnancy Childbirth. 2021 May 29;21:408. doi: 10.1186/s12884-021-03880-9 (PMC8164300; doi:10.1186/s12884-021-03880-9)
Supplement: Supplementary file 1 — Additional file 1. ANC Score and Indicator Matrix [file 12884_2021_3880_MOESM1_ESM.pdf]

ANC Element (Composite)	Input Indicators		Input Variables (Items)
**Readiness**	**ANC Structural Requirements**		operational equipment sterilistaion unit<br>operational laboatoy unit<br>operational pharmacy/dispensary operational
	**ANC Staff & Equipment**		at least 2 qualified ANC providers<br>functional blood pressure device<br>functional thermometer
	**ANC Medications & Supplies**		sulfadoxine-pyrimethamine tablets in stock<br>Tetanus vaccine (TT) in stock<br>iron folate OR iron PLUS folate tablets in stock<br>insecticide-treated bednets in stock
	**ANC Diagnostic Tests**		rapid hemoglobin tests in stock<br>urine protein testing strips in stock<br>rapid HIV tests in stock<br>rapdi RPR tests in stock
**Screening first visit cases**	**ANC-focused History**	**Obstetric History**	date od first day LMP assessed<br>previous pregnancies (gestations) assessed<br>previous uncomplicated pregnancies/deliveries assessed<br>previous c-sections assessed<br>previous premature birth assessed<br>previous stillbirths assessed<br>previous miscarriages assessed
		**Pregnancy History**	occurrence of bleeding assessed<br>occurrence of abdominal pain assessed<br>occurrence of breathing difficulties assessed<br>occurrence of headache/blurred visions assessed<br>occurrence of convulsion assessed<br>occurrence of nausea/vomiting assessed
		**Medical History**	pre-existing hypertension assessed<br>pre-existing diabetes assessed<br>history of TB assessed<br>history of STIs assessed<br>HIV status assessed
		**Medico-Social History**	medication use assessed<br>mental health / stress assessed<br>exposure to domestic violence assessed
	**ANC-focused Exam**	**Vital Sign Assessment**	blood pressure checked<br>other vital signs (pulse OR temperature OR respiratory rate)<br>fetal heart rate checked
		**Physical Examination**	conjunctiva checked for anemia<br>lower extremities checked for edema<br>abdomen/uterus checked<br>hand hygiene performed prior to exam
		**Diangostic Testing**	HIV test initiated (if unknown status)<br>blood test for RPR (syphilis) initiated<br>hemoglobin level checked
**Screening follow-up cases**	**ANC-focused History**	**Obstetric History**	antenatal record reviewed for previously documented obstetric history
		**Pregnancy History**	occurrence of bleeding assessed<br>occurrence of abdominal pain assessed<br>occurrence of breathing difficulties assessed<br>occurrence of headache/blurred visions assessed<br>occurrence of convulsion assessed<br>occurrence of nausea/vomiting assessed
		**Medico-Social History**	medication use assessed<br>mental health / stress assessed

			exposure to domestic violence assessed
	**ANC-focused Exam**	**Vital Sign Assessment**	blood pressure checked<br>other vital signs (pulse OR temperature OR respiratory rate)<br>fetal heart rate checked
		**Physical Examination**	conjunctiva checked for anemia<br>lower extremities checked for edema<br>abdomen/uterus checked<br>hand hygiene performed prior to exam

**Prevention first visit cases**	**Prescription or administration**	iron folate prescription<br>patient instructed on follow-up iron folate refill<br>IPTp correctly administered in respect to trimester<br>patient instructed on follow-up IPTp treatment<br>TT administred (once status checked)<br>insecticide-treated bednet handed out
**Prevention follow-up cases**	**Prescription or administration**	iron folate prescription<br>patient instructed on follow-up iron folate refill<br>IPTp correctly administered in respect to trimester<br>patient instructed on follow-up IPTp treatment<br>TT administred (once status checked)

**Information & Education**	**Info/Education related to pregnancy**	results of examination/testing explained or discussed<br>patient is given opportunity for questions<br>education on safety / social support<br>education on diety & nutrition<br>education on use of insecticide-treated bednet<br>education on health seeking in respect to danger signs
	**Info/Education related to birth**	education on birth companion<br>education on birth & emergency preparedness plan<br>education on breastfeeding<br>education on postpartum family planning
